# From Anhydrous Zinc Oxide Nanoparticle Powders to Aqueous Colloids: Impact of Water Condensation and Organic Salt Adsorption on Free Exciton Emission

**DOI:** 10.1021/acs.langmuir.9b00656

**Published:** 2019-06-21

**Authors:** Krisztina Kocsis, Matthias Niedermaier, Vít Kasparek, Johannes Bernardi, Günther Redhammer, Michel Bockstedte, Thomas Berger, Oliver Diwald

**Affiliations:** † Department of Chemistry and Physics of Materials, University of Salzburg, Jakob-Haringer-Strasse 2a, 5020 Salzburg, Austria; ‡ Central European Institute of Technology, Brno University of Technology, Purkynova 123, 612 00 Brno, Czech Republic; § University Service Centre for Transmission Electron Microscopy, Technische Universität Wien, 1040 Vienna, Austria

## Abstract

Variations in the composition and structure of ZnO nanoparticle interfaces have a key influence on the materials’ optoelectronic properties and are responsible for high number of discrepant results reported for ZnO-based nanomaterials. Here, we conduct a systematic study of the room-temperature photoluminescence of anhydrous ZnO nanocrystals, as synthesized in the gas phase and processed in water-free atmosphere, and of their colloidal derivatives in aqueous dispersions with varying amounts of organic salt admixtures. A free exciton band at *hv* = 3.3 eV is essentially absent in the anhydrous ZnO nanocrystal powders measured in vacuum or in oxygen atmosphere. Surface hydration of the nanoparticles during colloid formation leads to the emergence of the free exciton band at *hv* = 3.3 eV and induces a small but significant release in lattice strain as detected by X-ray diffraction. Most importantly, admixture of acetate or citrate ions to the aqueous colloidal dispersions not only allows for the control of the ζ-potential but also affects the intensity of the free exciton emission in a correlated manner. The buildup of negative charge at the solid—liquid interface, as produced by citrate adsorption, increases the free exciton emission. This effect is attributed to the suppression of electron trapping in the near-surface region, which counteracts nonradiative exciton recombination. Using well-defined ZnO nanoparticles as model systems for interface chemistry studies, our findings highlight water-induced key effects that depend on the composition of the aqueous solution shell around the semiconducting metal oxide nanoparticles.

## Introduction

Synthesis and colloidal processing of semiconducting oxide nanostructures matter once their optical and electronic properties depend on their interfaces. This is largely caused by the electronic influence of surfactants, additives, and solvents, which make up the dynamic solid—liquid interface.^1–5^ Surfactants are regularly employed as capping agents to enable anisotropic growth during metal oxide synthesis.^6,7^ Formulation strategies to generate nanocrystal dispersions for ceramics, thin-film deposition, and nanoparticle printing as examples require additives to adjust the dispersions’ rheological properties, to optimize the drying properties of nanoparticle-based pastes, and, last but not least, to integrate respective materials components into devices.^8,9^ Many of these operation steps involve continuous phase changes and lead to substantial modifications at the materials interfaces.

Nanostructured ZnO is widely used for catalysis, optical, and electronic applications. The still increasing interest in ZnO-based inorganic phosphors is clearly documented by the continuous rise in the number of publications related to the research topic ZnO nanoparticles and photoluminescence properties, as revealed by an up-to-date literature survey using Web of Science or other databases. A substantial body of work has established robust connections between photoluminescence excitation and emission properties of ZnO structures and different defect types contained therein.^10–25^ Although many optically and electronically active defects seem to be linked to the interfaces of ZnO nanostructures, systematic studies on the impact of nature and composition of the interface on the photoluminescence properties are scarce.^1,11,26–28^

During colloidal processing of ZnO nanoparticles,^29^ surfactant molecules and polymers are adsorbed from solution onto the particles surfaces.^2,8,30,31^ The adsorption of citrate or acetate ions, for instance, changes the ζ-potential values of the particle dispersions, enables one to increase electric doublelayer repulsion for the stabilization of colloidal dispersions,^32^ and affects the free exciton emission intensity.^33^ Changes in the broad defect-related luminescence band are reported for other inorganic or organic adsorbates.^34–37^ Apart from a number of studies that focus on the impact of specific molecules,^38–41^ a general understanding of how adsorbed surfactants and the solvents’ dielectric properties can affect energy and intensity of photoluminescence bands is far from complete.

With this very first systematic comparison of the free exciton emission of ZnO nanocrystals, which were grown in the gas phase, processed, and measured under water-free conditions, with identical nanoparticles in contact with a condensed water phase, we discuss important optical property changes that can originate from the conversion of the anhydrous powder into an aqueous colloidal dispersion. The present investigation connects to a previously performed study on aqueous ZnO colloids that aimed at the relationship between adsorption-induced photoluminescence property changes and the yield of photogenerated charges that are accessible to electron paramagnetic resonance spectroscopy.^33^ Here, we used two widely employed surfactants, citrate ions producing negative ζ-potential values, and acetate ions, the adsorption of which shifts the corresponding ζ-potential values to more positive values. We will demonstrate that the corresponding trends in the intensity of the free exciton emission at λ = 380 nm (*hv* = 3.3 eV), which is absent on dehydroxylated ZnO nanoparticle powders, show a clear dependence on the ζ-potential in aqueous dispersion. As an important aspect of our investigation, we included the colloidal stability of the ZnO nanoparticle dispersion for the discussion of the spectroscopic data and were able to exclude artifacts that arise from aggregation and sediment formation. The observed variations in exciton emission intensity are explained by local polarization changes originating from acetate and citrate ions in combination with their very different molecular dipole moments. Highlighting the importance of interfacial properties of semiconductor nanoparticles with regard to a robust description of their optoelectronic properties, these findings should contribute to more reproducible colloidal manufacturing protocols of ZnO-containing optoelectronic devices, functional coatings, and polymer nanocomposites.

## Experimental Section

ZnO nanoparticles were produced by metal-organic chemical vapor synthesis using zinc acetate dihydrate (Sigma-Aldrich, ≥99.0%) as a precursor.^42^ An optimized sequence of alternating vacuum annealing and oxidation steps was applied to the powder sample for removal of synthesis-related organic impurities: high vacuum conditions at *T* = 473 K (heating rate: 2.5 K/min, dwell time: 1 h), followed by oxidative treatment in O_2_ at *T* = 573 K (heating rate: 5 K/min, dwell time: 30 min) and 673 K (heating rate: 5 K/min, dwell time: 1 h), which ultimately leads to stoichiometric ZnO nanoparticles with a level of residual surface carbon of less than 5%.^42,43^

Size distribution functions in colloidal samples and ζ-potential values of the secondary particles were determined for aqueous dispersions with a particle concentration of 0.1 mg/mL. For their preparation, water with a resistivity of 18 MΩ cm was added to ZnO nanoparticles. Trisodium citrate dihydrate (Merck, ≥99.0%) or zinc acetate dihydrate (Sigma-Aldrich, ≥99.0%) were used as organic salts. First, the stock solutions of the salts were prepared and added to the particles—water mixture in different concentrations relative to the particle mass. The pH values recorded for all of these dispersions were in the range of 7.0-8.1 and slightly increased with the concentration of citrate added. To complement mechanical stirring under cooling in an ice bath, we also employed direct ultrasound irradiation of the dispersion using an ultrasonic finger (15 min, amplitude 25%, UP200St, Ti-sonotrode Ø 2 mm, Hielscher Ultrasonics).

**Figure 2:**
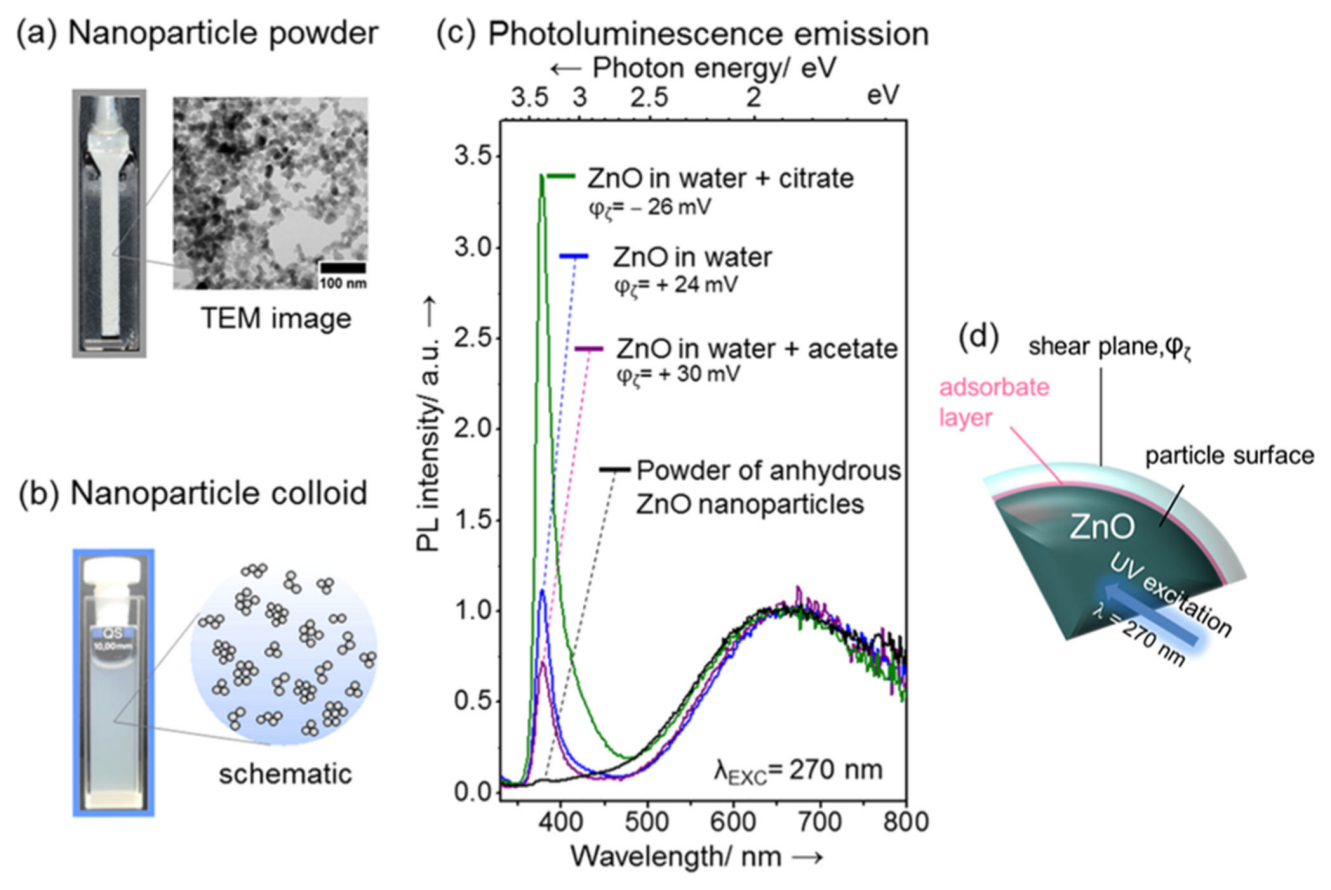
Anhydrous powders of ZnO nanoparticles in vacuum (a) and aqueous ZnO colloids derived therefrom (b), showing different photoluminescence emission intensities at λ = 380 nm. (c) Citrate and acetate salt addition increases and decreases the intensity of the excitonic emission, respectively, and changes the ζ-potential value in an opposite manner. (d) Scheme outlining that different regions of the ZnO nanoparticle interfaces are probed with photoluminescence spectroscopy and ζ-potential measurements. The citrate and acetate concentrations in the aqueous dispersions were 50 and 30 w/w % (relative to particle mass), respectively.

**Figure 3:**
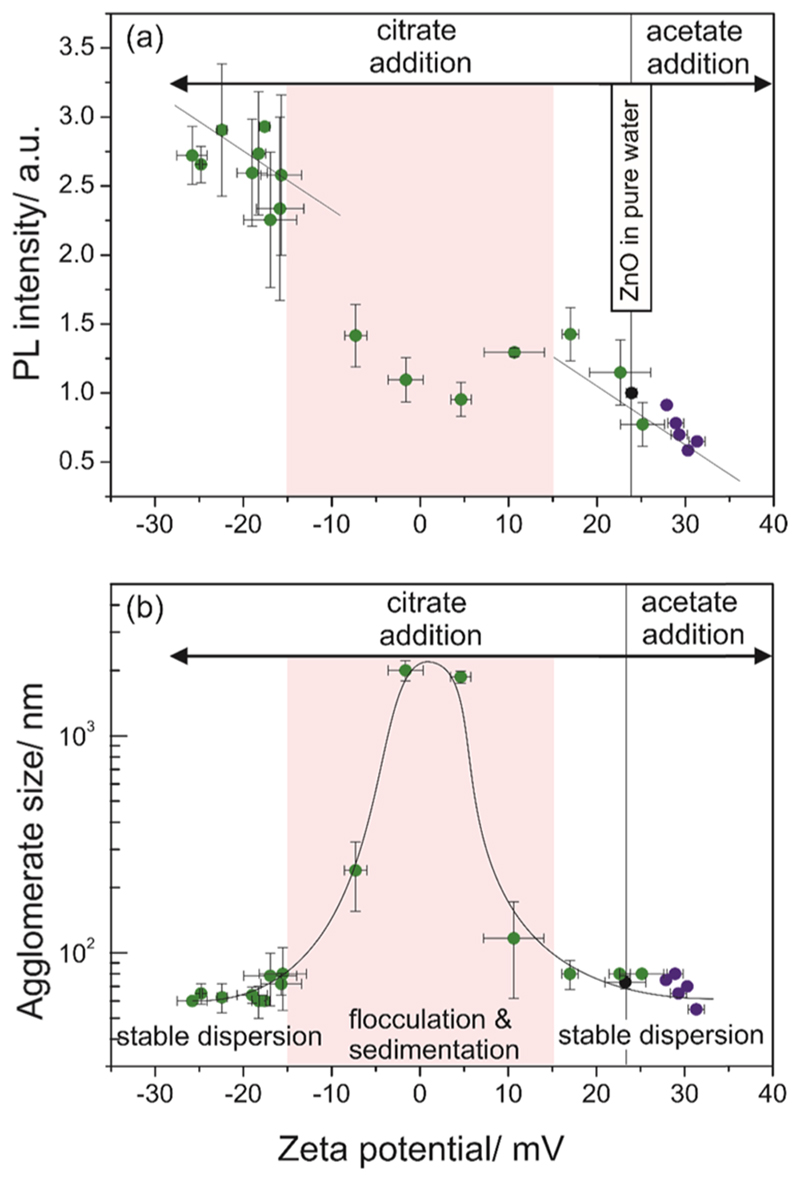
Plot of photoluminescence emission intensities related to the excitonic band at λ = 380 nm (a) and the particle size (b) against ζ-potential values measured for aqueous colloidal dispersions of different citrate or acetate contents. As demonstrated in (b), the intermediate range of ζ-potentials, i.e., for values in the range of —15 mV < φ_ζ_ < +15 mV, describes unstable colloidal dispersions. A compilation of salt concentrations versus hydrodynamic radii and ζ-potential values is provided by Tables S2 and S3 of the Supporting Information. For all experiments, the pH values were in the range of 7—8.

Both dynamic and electrophoretic light scattering measurements were performed on Zetasizer Nano ZSP ZEN5600 (Malvern Instruments), which operates with red laser light (λ = 632.8 nm). For size distribution curves, the scattering information was collected in backscatter mode with a detection angle of 175°. The Smoluchowski approximation was used to determine the sample’s ζ-potential from the electrophoretic mobility of the particles at an applied voltage of 40 V. Photoluminescence measurements were carried out with a double-grating PL spectrometer system FLS980 (Edinburgh Instruments). Two different measurement setups were used for colloids and powders, i.e., the standard right angle geometry for liquid colloidal dispersions and a front-facing sample holder suitable for powder samples.^43^ We did not normalize the PL emission spectra related to the colloidal samples (Figures 2 and 3) but magnified the one and only powder spectrum shown in Figure 2 to a value that allows for qualitative comparison between the powder and the colloid spectra.

Particle specimen from powders and colloidal dispersions were analyzed by transmission electron microscopy (TEM) using a TECNAI F20 field emission instrument. The electron microscopic and analytical measurements were obtained on the material sticking to the TEM grid either after dipping a lacey carbon grid into the powder or by putting a droplet of the dispersion on the grid followed by drying in air. Electron micrographs were recorded using a Gatan Orius CCD camera. The size distribution of individual particles was derived from the analysis of TEM data by counting between 200 and 300 particles for each sample. The error that arises from overlapping particles or particle contacts with insufficient contrast is below the value of the size increments plotted along the x-axis of the particle size distribution plots. On the basis of the observation of all of the sample spots investigated, we can exclude any prevailing type of particle shape anisotropy. For this reason, the majority of ZnO nanoparticles investigated here, either directly from analyzing the particles of the powder or after subsequent transformation into a colloid, can be characterized as equiaxed grains and are approximated as spheres.

For crystallite size and microstrain determination, step-scan powder X-ray diffraction (XRD) data were collected at room temperature in coupled *θ-θ* mode on a Bruker D8 Advance DaVinci-Design diffractometer, having a goniometer radius of 280 mm and being equipped with a fast solid-state Lynxeye detector. Data acquisition was done using Cu Kα_1,2_ radiation between 2θ =15 and 130°, with a step size of 0.01° and integration time of 1 s, with the divergence slit and the receiving slit opened at 0.3 and 2.5°, respectively; a primary and secondary side 2.5° Soller slit was used to minimize axial divergence, and the detector window opening angle was chosen as 2.95°. Data handling was done with TOPAS 4.2 (Bruker 2012) using whole pattern refinement and a double-Voigt approach.^44^ The intrinsic peak shape of the Bragg peaks was modeled with the fundamental parameter approach. The crystallite size broadening was then handled by allowing a Lorentzian type, while microstrain was handled by a Gaussian-type component convolution.

## Results and Discussion

**Figure 1:**
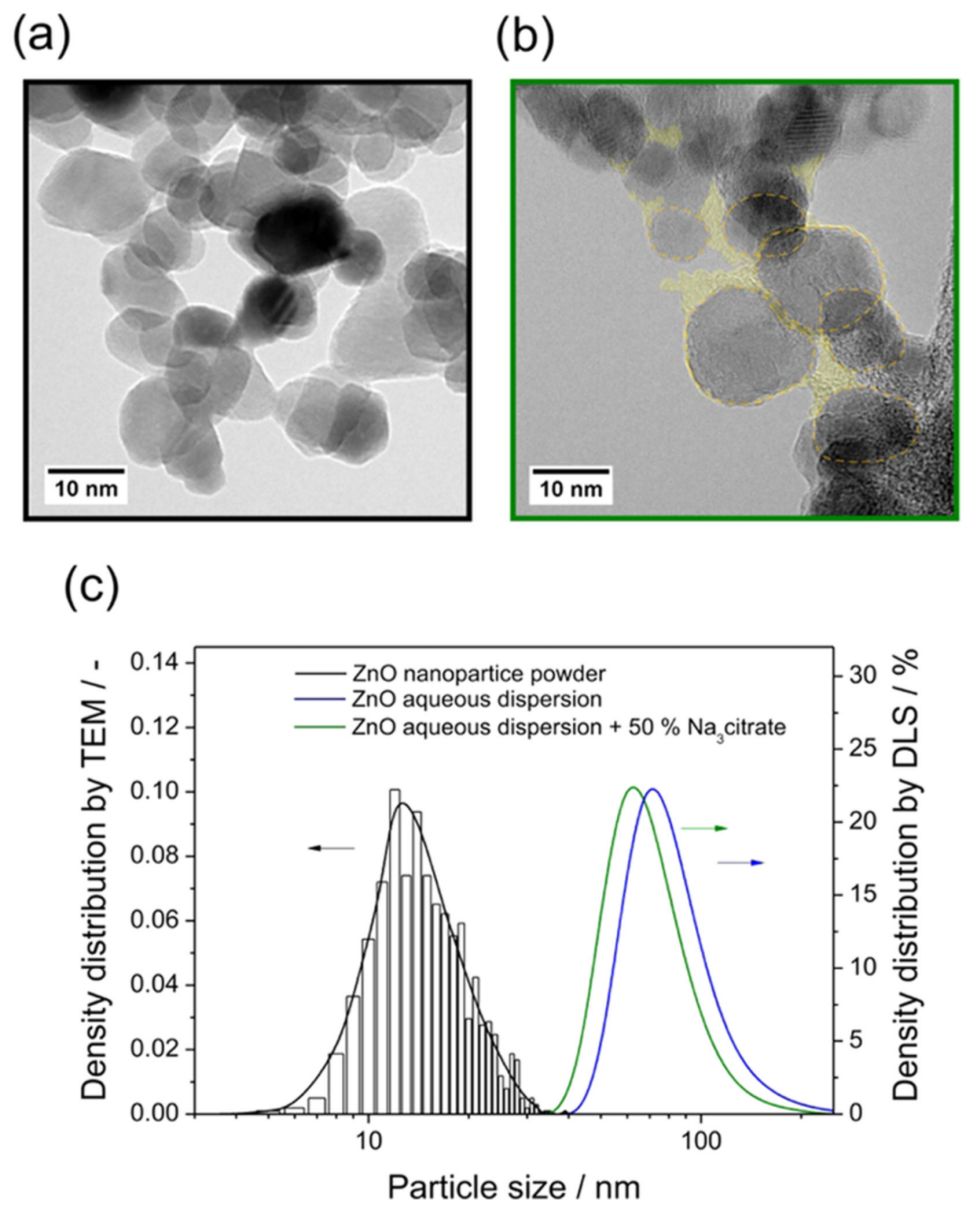
TEM images of vapor-phase-grown ZnO nanoparticles (a) before and (b) after contact with an aqueous citrate solution. The yellow region in (b) indicates amorphous and carbon-based surface features around the ZnO nanoparticles originating from adsorbed organics after vacuum drying and electron beam damage. (c) Particles size distribution plots (bar diagram from TEM analysis and curves from dynamic light scattering (DLS) measurements).

After gas-phase synthesis and thermal processing in water-free gas atmospheres, i.e., the application of alternating cycles of sample treatment in vacuum or in dry oxygen, the ZnO nanoparticle powders are characterized by a narrow particle size distribution peaking at d_TEM_ = 14 nm (Figure 1a,c). The average crystalline domain size was determined to be d_XRD_= 10 nm as calculated from the Scherrer equation. Prior to the water adsorption experiments, which will be described below, the ZnO nanoparticle surfaces are free from adsorbed solvent molecules, inorganic ions, surfactants, and other synthesis-related remnants. (An earlier Auger electron spectroscopy study revealed residual carbon species with a surface concentration of up to 5% as the only impurity present.^42,43^)

The yellow region in Figure 1b highlights amorphous and carbon-based surface features around the ZnO nanoparticles, which result from the adsorbed organics after vacuum drying and electron beam damage. They were observed for all analyzed sample spots of the citrate-functionalized nanoparticles, but were not observed in samples that were exclusively processed in vacuum and oxygen. (Unprocessed images for reference and comparison are shown in Figure S1 of the Supporting Information.)

Once aqueous ZnO nanoparticle dispersions with particle concentrations of 0.1 mg/mL were prepared and showed stability during investigation and for a minimum of 2 h, the particle size distribution functions were additionally determined by dynamic light scattering (DLS). The size distribution plots show maxima below 100 nm and characterize the nanoparticle agglomerates (or secondary particles) inside the colloidal dispersion. In the following, we will refer to the individual particles as primary particles or just particles as part of these agglomerates.

The condensation and adsorption of water molecules at the ZnO nanocrystal surfaces lead to the emergence of an excitonic emission at λ = 380 nm (blue curve in Figure 2), which is not observed for vapor-phase-grown ZnO nanoparticles (black curve in Figure 2).^43^ During the dispersion of the dry ZnO nanoparticle powder into an aqueous phase, a water solvation shell is built up around the nanoparticles and concomitantly a ζ-potential develops. This ζ-potential, as the electrical potential at the shear plane of the particle, was determined by electrophoretic measurements (inset in Figure 2d). Consistent with earlier results on identical materials^43^ and also in good agreement with ZnO materials of different synthetic origin but comparable particle size and structure,^45^ we determined for the nanoparticles a ζ-potential value of +24 mV (Figure 2c). Adsorption of potential determining ions modifies the charge distribution at the interface, the ζ-potential, and consequently the stability of particle dispersions. While the addition of 30 w/w % acetate (relative to particle mass) produces a shift of the ζ-potential toward more positive values, we observe an inversion of the ζ-potential value to φ_ζ_= -26 mV upon addition of 50 w/w % citrate. Related changes result from the adsorption of potential determining ions and from protonation—deprotona-tion reactions at the solid—liquid interface due to pH changes. In parallel to the adsorption-induced changes in the ζ-potential, we also observed a change in the PL emission band intensity related to the radiative deactivation of the free exciton; while citrate produces an intensity increase of the excitonic emission at λ = 380 nm, acetate addition leads to a decrease (Figure 2c). There exist a number of reports in the literature that describe the dependence of PL emission features in the visible light range on adsorption.^34—37^ A correlation of the ζ-potential with the visible light emission based on admixture of inorganic salts to nonpolar and aqueous solvents was reported,^35^ where the excitonic emission (albeit blue-shifted) remained essentially unchanged. Note that the interaction of the inorganic and organic adsorbates employed in the above works with the ZnO particle surface is different from the adsorption of citric acid and acetate used in our work. In the present case, namely, there is no correspondence between adsorbate-induced intensity changes related to the free exciton emission at λ = 380 nm and the broad emission band centered at λ = 670 nm, the intensity of which remains constant throughout all of the experiments performed for this study and which therefore is also not correlated with the observed ζ-potential changes. Hence, under the experimental conditions, applied citrate and acetate adsorption have no significant influence on potential energy transfer steps that convert a fraction of the exciton energy into a defect-related emission in the visible light range.^33^

In parallel to solvent-induced changes in the free exciton emission, we also looked for potential changes in the crystallinity of the nanocrystals. For this purpose, we performed laboratory XRD measurements and subjected the acquired diffraction patterns to refinement using the double-Voigt approach.^44^ As crystallite size and microstrain convolutions vary in 2*θ* as a function of 1/cos(*θ*) and tan(*θ*), one can separate these contributions from each other with data that are acquired up to sufficiently high 2*θ* angles. The as- synthesized nanoparticles, the PL emission properties of which were studied in a previous study,^42^ exhibit the largest microstrain. Subsequent thermal powder activation leads to its relaxation from *ε* = 0.385(10) to 0.225(6) (Table S1 in the Supporting Information). While extended air exposure (over a period of ∼7 weeks) does not affect the sample’s average crystallite size and the microstrain within the margin of the estimated standard deviations, ZnO nanoparticle powders in contact with liquid water (with or without organic acids) exhibit a further reduction in microstrain (Supporting Information Figure S2 and Table S1). This reduction in strain from ε = 0.225(6) to 0.198(4) is observed for thermally activated samples, which serve here as a starting material, and upon contact with condensed H_2_O. Although low in value, the extent of strain reduction exceeds the estimated standard deviation by a factor of 4. This also underlines that the nanoparticles experience substantial changes of their chemical environment when they convert from dry nanoparticle powders into aqueous nanoparticle dispersions. It is remarkable that such strain relaxation effects are measurable for the investigated nanoparticles in the size range of 10—20 nm (Figure 1c). For these, as compared to colloidal quantum dots, comparatively large nanoparticles, only a small fraction of the total number of ions of the particle is part of the surface and the near-surface region, where relaxation effects occur in response to surrounding phase changes. The bulk fraction of particle ions, which predominantly contribute to the measured signal, however, is expected to be decoupled from compositional and structural changes at the particle surface. Apparently, the surface and near-surface layers remain distorted even after thermal activation,^46,47^ whereas the interior of the nanoparticles retains the wurzite structure. As a result of contact with liquid water, surface ions with a lack of local coordination partners adsorb water molecules and the associated partial relaxation of strain^48^ gives rise to improved crystallinity.^49^ At the same time, water adsorption enhances the probability of radiative exciton annihilation.

In addition to and associated with the observed relaxation of lattice strain, it is the chemical composition of the solid—liquid interface, in particular the ligand shell around the ZnO nanoparticles, that determines the photoluminescence emission yield (Figure 3a) and the ζ-potential (Figure 3b). Figure 3 illustrates how the addition of citrate and acetate salts to aqueous dispersions with identical nanoparticle loadings affect the intensity of the free exciton emission toward more negative and positive values, respectively. Despite the scattering of the data points, there is a clear trend in the ζ-potential regimes of φ_ζ_ < -15 mV and φ_ζ_ > +15 mV.

In the regime -15 mV ≤φ_ζ_ ≤ +15 mV, the ζ-potential exhibits a strong dependence on concentration in the range below 8 w/w-% citrate relative to the particles’ mass. For a citrate concentration of 3 w/w-%, we measured a ζ-potential of 0 mV and—due to the loss of electrostatic stabilization—an increase of the hydrodynamic diameter of particle agglomerates from 70 to 2000 nm (Figures 3b and S3 of the Supporting Information). This concentration corresponds to about 500 molecules dissolved and/or adsorbed per particle. Even in the hypothetic case of complete and irreversible citrate adsorption, a fraction of 30% of a nanoparticle surface would be covered. A time-averaged composition of the interfacial layer, which, at present, we cannot describe qualitatively and quantitatively, evolves as a result of the dynamic interaction between dissolved molecules and dispersed ZnO nanoparticles. The complexity of interface structure and composition is further increased by the fact that organic molecules displace surface-adsorbed hydrogen, which again affects partial surface charges and local surface dipole moments. In the absence of citrate ions respective hydrogen species yield at the nanoparticle surface a ζ-potential of +24 mV and stabilize the ZnO nanoparticle-based colloids against agglomeration. By increasing the citrate concentration beyond the isoelectric point, the ζ-potential adopts more negative values and the colloidal dispersion regains stability as the hydrodynamic agglomerate diameters decrease to *d_h_* = 60 nm (Figure 3b).

Furthermore, the addition of acetate ions shifts the ζ-potential to more positive values and, hence, maintains the stability of the aqueous colloidal dispersion. The strongly diminished electric double-layer repulsion in the ζ-potential range —15 mV < φ_ζ_ < +15 mV explains the observed metal oxide nanoparticle flocculation and sedimentation, which is also visible to the naked eye and which does not allow for a reproducible assessment of the photoluminescence emission properties during the time intervals typically used for the photoluminescence measurements.

**Figure 4:**
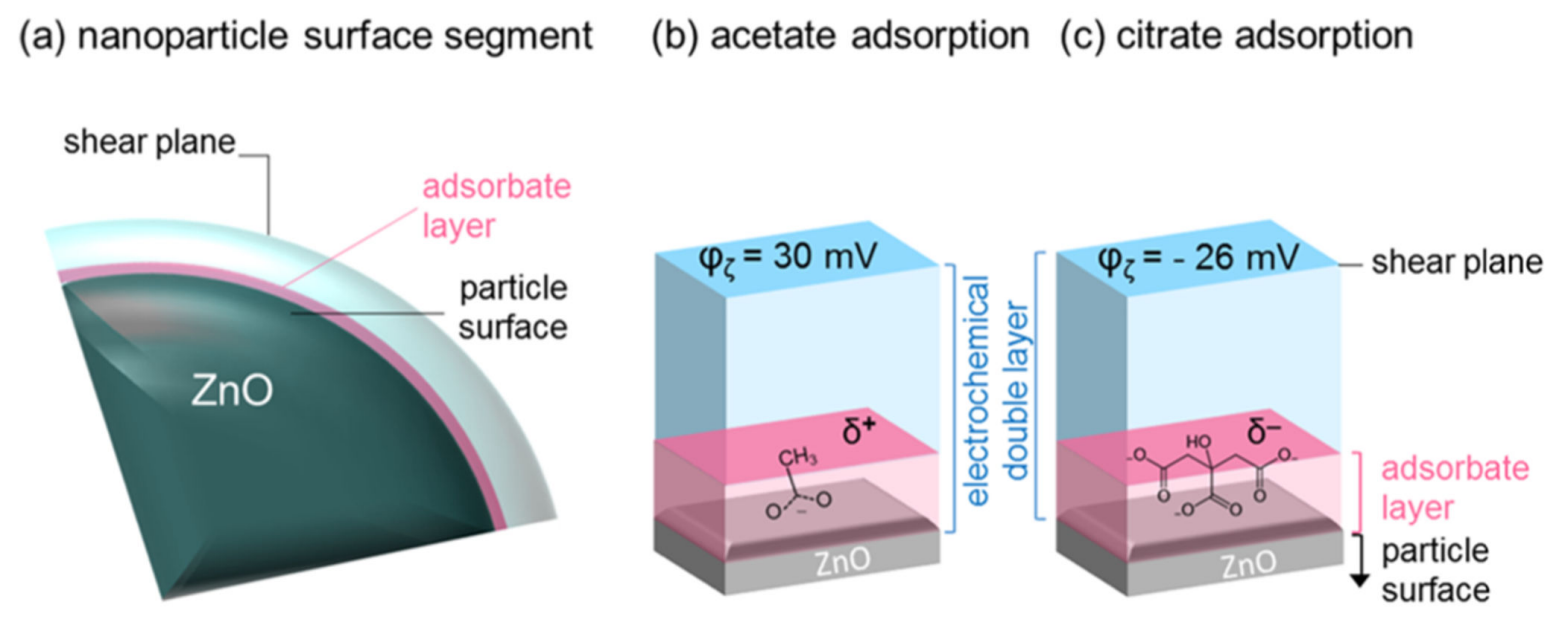
Schematic illustration of the structure of the different types of surface layers around a ZnO nanoparticle being surrounded by an adsorbate layer of organic ions and a bulk condensed aqueous-phase liquid above.

Composition and structure of the water shell around the colloidal nanoparticle (Figure 4a) and the thickness of the electrochemical double layer corresponding to the distance between the particle surface (Figure 4b,c), with an essentially unknown surface potential, and the position of the shear plane at which the ζ-potential can be probed are complex and subject to dynamic changes.^40,50,51^ Among others, material parameters like ZnO surface coverage with different adsorbate species, adsorbate geometry, and composition and thickness of the electrochemical double layer are yet unknown.

Nevertheless, as we argue in more detail below, we expect a preferential adsorption of acetate or citrate ions via their carboxyl groups generating locally either a positively or negatively charged adsorbate layer (Figure 4b,c). At the same time, both types of ions possess with their carboxylic linkers chemically identical anchor groups that attach to the surface Zn^2+^ ions where they displace hydrogen species. The functional dependence of the PL intensity of the free exciton emission on the ζ-potential is clearly related to the adsorption of these species at the ZnO nanoparticle surface.

For a further analysis, we make reference to experiments describing water adsorption on well-defined and atomically clean single crystal surfaces: combined experimental and theoretical investigations^52–54^ revealed that adsorbed hydrogen species have a strong coverage-dependent effect on the local work function and the doping level of the semiconductor structure. The concomitant localization of excess charge carriers at the surface increases the probability for exciton recombination. These important observations suggest that the replacement of any hydrogen-related species by acetate or citrate ions at the nanoparticle surfaces must also affect the local potentials and, hence, the probability for radiative exciton recombination.

The reported hydrogen concentrations in the bulk of ZnO single crystals^55—57^ suggest a full depletion of nanoparticles in the size regime between 1 and 25 nm (Figure 1). This emphasizes the role of local potential effects at the surface rather than effects due to longer-ranged band bending,^21,35,36^ which are neglected for the here investigated nanoparticle ensembles. The local effect caused by adsorption of hydrogen species is furthermore suggested by the suppression (extinction) of the exciton peak in the anhydrous ZnO nanoparticle powders (Figure 2) and its appearance upon nanoparticle immersion into pure water. For nanoparticles with bare surfaces, nonradiative recombination can occur at surface dangling bonds or other types of defects that are associated with coordinatively unsaturated surface ions.^24^ Related effects of the built-in potential suppress the radiative recombination of free excitons compared to the particles in water, where local potential effects and surface doping increase the PL intensity.^52,53^

As discussed above, acetate and citrate ions have their identical carboxylic anchor group, which displaces surface hydrogen species, in common. On the other hand, the two molecules give rise to opposite effects in photoluminescence emission and in the direction of the φ_ζ_ development as a function of the salt concentration (Figure 3). This can be rationalized in the following way: the methyl rest of the acetate group carries a positive partial charge (Figure 4b), adsorbed citrate species exhibit—depending on whether the adsorption complex adopts a mono- or bidentate configuration—one or two negatively charged carboxylic moieties, respectively. These are oriented away from the ZnO surface and toward the bulk electrolyte solution (Figure 4b,c). We speculate that the very different molecular dipole of the adsorbed citrate ions together with their contribution to the local polarization at the ZnO electrolyte interface favors the delocalization of excess charge carriers and, hence, enhances the probability for radiative exciton recombination. Conversely, acetate adsorption reduces the density of charge carriers in the surface region and thereby the probability for radiative exciton recombination.

## Conclusions

For the first time, we compared the room-temperature photoluminescence emission properties of vapor-phase-grown ZnO nanocrystals in water-free environments to those of identical particles but transformed into aqueous colloids. In addition to water adsorption-induced release of lattice strain in the near-surface region, we also observed that water adsorption promotes the radiative deactivation of the free exciton emission at *hv* = 3.3 eV. After addition of dissolved citrate or acetate ions to the aqueous dispersion, the ζ-potential values shift to more negative or more positive values, respectively. The trends in the optoelectronic properties observed show a functional dependence on the ζ-potential. They are rationalized by the adsorption of organic ions at the ZnO nanoparticle surfaces where they displace hydrogen species and, hence, affect their local potential, whereas the different molecular dipoles affect the probability for radiative exciton recombination. Related insights are of key importance for the fabrication of ZnO-containing devices, for functional coatings and polymer nanocomposites, where reproducible optoelectronic properties are a necessary requirement but subject to changes during processing.

## Supplementary Material

The Supporting Information is available free of charge on the ACS Publications website at DOI: 10.1021/acs.lang-muir.9b00656.

Additional and unprocessed TEM images of ZnO nanoparticles from aqueous dispersions with and without adsorbed organic ions, and details of the microstrain analysis and particle size distribution (PDF)

Supplementary information

## References

[1] Boles MA, Ling D, Hyeon T, Talapin DV (2016). The Surface Science of Nanocrystals. Nat Mater.

[2] Distaso M, Bertoni G, Todisco S, Marras S, Gallo V, Manna L, Peukert W (2017). Interplay of Internal Structure and Interfaces on the Emitting
Properties of Hybrid ZnO Hierarchical Particles. ACS Appl Mater Interfaces.

[3] Heinz H, Pramanik C, Heinz O, Ding Y, Mishra RK, Marchon D, Flatt RJ, Estrela-Lopis I, Llop J, Moya S, Ziolo RF (2017). Nanoparticle Decoration with Surfactants: Molecular Interactions,
Assembly, and Applications. Surf Sci Rep.

[4] Shen Z, Chun J, Rosso KM, Mundy CJ (2018). Surface Chemistry Affects the Efficacy of the Hydration Force
between Two ZnO(1010) Surfaces. J Phys Chem C.

[5] Zobel M, Neder RB, Kimber SAJ (2015). Universal Solvent Restructuring induced by Colloidal
Nanoparticles. Science.

[6] Zhang T, Dong W, Keeter-Brewer M, Konar S, Njabon RN, Tian ZR (2006). Site-specific Nucleation and Growth Kinetics in hierarchical
Nanosyntheses of Branched ZnO Crystallites. J Am Chem Soc.

[7] Zhang X, Shen Z, Liu J, Kerisit SN, Bowden ME, Sushko ML, Yoreo JJde, Rosso KM (2017). Direction-specific Interaction Forces underlying Zinc Oxide
Crystal Growth by Oriented Attachment. Nat Commun.

[8] Franks GV, Tallon C, Studart AR, Sesso ML, Leo S (2017). Colloidal Processing: Enabling Complex Shaped Ceramics with
unique Multiscale Structures. J Am Ceram Soc.

[9] Pillai SC, Kelly JM, McCormack DE, O’Brien P, Ramesh R (2003). The Effect of Processing Conditions on Varistors prepared from
Nanocrystalline ZnO. J Mater Chem.

[10] Choi S, Phillips MR, Aharonovich I, Pornsuwan S, Cowie BCC, Ton-That C (2015). Photophysics of Point Defects in ZnO
Nanoparticles. Adv Opt Mater.

[11] Stavale F, Nilius N, Freund H-J (2013). STM Luminescence Spectroscopy of Intrinsic Defects in ZnO(0001)
Thin Films. J Phys Chem Lett.

[12] Stavale F, Pascua L, Nilius N, Freund H-J (2014). Luminescence Properties of Nitrogen-doped ZnO. J Phys Chem C.

[13] Klingshirn C, Fallert J, Zhou H, Sartor J, Thiele C, Maier-Flaig F, Schneider D, Kalt H (2010). 65 Years of ZnO Research - Old and very Recent
Results. Phys Status Solidi B.

[14] Djurisić AB, Leung YH (2006). Optical Properties of ZnO Nanostructures. Small.

[15] van Dijken A, Meulenkamp EA, Vanmaekelbergh D, Meijerink A (2000). Identification of the Transition Responsible for the Visible
Emission in ZnO Using Quantum Size Effects. J Lumin.

[16] Ischenko V, Polarz S, Grote D, Stavarache V, Fink K, Driess M (2005). Zinc Oxide Nanoparticles with Defects. Adv Funct Mater.

[17] Janotti A, van de Walle CG Native Point Defects in ZnO. Phys Rev B.

[18] Brauer G, Anwand W, Grambole D, Egger W, Sperr P, Beinik I, Wang L, Teichert C, Kuriplach J, Lang J, Zviagin S (2009). Characterization of ZnO Nanostructures: A Challenge to Positron
Annihilation Spectroscopy and other Methods. Phys Status Solidi C.

[19] Wang D, Chen ZQ, Wang DD, Qi N, Gong J, Cao CY, Tang Z (2010). Positron Annihilation Study of the interfacial Defects in ZnO
Nanocrystals: Correlation with Ferromagnetism. J Appl Phys.

[20] Knutsen KE, Galeckas A, Zubiaga A, Tuomisto F, Farlow GC, Svensson BG, Kuznetsov AY (2012). Zinc Vacancy and Oxygen Interstitial in ZnO revealed by
sequential Annealing and Electron Irradiation. Phys Rev B Condens Matter Mater Phys.

[21] Zhang Z, Yates JT (2012). Band Bending in Semiconductors: Chemical and Physical
Consequences at Surfaces and Interfaces. Chem Rev.

[22] Drouilly C, Krafft J-M, Averseng F, Lauron-Pernot H, Bazer-Bachi D, Chizallet C, Lecocq V, Costentin G (2013). Role of Oxygen Vacancies in the Basicity of ZnO: From the Model
Methylbutynol Conversion to the Ethanol Transformation
Application. Appl Catal, A.

[23] Zhang H, Gheisi AR, Sternig A, Müller K, Schowalter M, Rosenauer A, Diwald O, Mädler L (2012). Bulk and Surface Excitons in Alloyed and Phase-Separated ZnO-MgO
Particulate Systems. ACS Appl Mater Interfaces.

[24] Berger T, Diwald O, Jupille J, Thornton G (2015). Defects at Oxide Surfaces.

[25] Stankic S, Sternig A, Finocchi F, Bernardi J, Diwald O (2010). Zinc Oxide Scaffolds on MgO Nanocubes. Nanotechnology.

[26] Idriss H, Andrews RM, Barteau MA (1993). Application of Luminescence Techniques to Probe Surface-Adsorbate
Interactions on Oxide Single Crystals. J Vac Sci Technol A.

[27] Idriss H, Barteau MA (1992). Photoluminescence From Zinc Oxide Powder to Probe Adsorption and
Reaction of O2, CO, H2, HCOOH, and CH3OH. J Phys Chem.

[28] Idriss H, Barteau MA (2000). Active Sites on Oxides: From Single Crystals to
Catalysts. Adv Catal.

[29] Gervasio M, Lu K (2019). Monte Carlo Simulation Modeling of Nanoparticle-Polymer
Co-Suspensions. Langmuir.

[30] Wu W (2017). Inorganic Nanomaterials for Printed Electronics: A
Review. Nanoscale.

[31] Bell NS, Monson TC, DiAntonio C, Wu Y (2016). Practical Colloidal Processing of Multication
Ceramics. J Ceram Sci Technnol.

[32] Hidber PC, Graule TJ, Gauckler LJ (1997). Influence of the Dispersant Structure on Properties of
Electrostatically stabilized Aqueous Alumina Suspensions. J Eur Ceram Soc.

[33] Kocsis K, Niedermaier M, Schwab T, Kasparek V, Berger T, Diwald O (2018). Exciton Emission and Light-Induced Charge Separation in Colloidal
ZnO Nanocrystals. ChemPhotoChem.

[34] Norberg NS, Gamelin DR (2005). Influence of Surface Modification on the Luminescence of
Colloidal ZnO Nanocrystals. J Phys Chem B.

[35] Ghosh M, Raychaudhuri AK (2008). Ionic Environment Control of Visible Photoluminescence from ZnO
Nanoparticles. Appl Phys Lett.

[36] Hodlur RM, Rabinal MK, Mohamed Ikram I (2014). Influence of Dipole Moment of Capping Molecules on the
Optoelectronic Properties of ZnO Nanoparticles. J Lumin.

[37] Sandmann A, Kompch A, Mackert V, Liebscher CH, Winterer M (2015). Interaction of L-Cysteine with ZnO: Structure, Surface Chemistry,
and Optical Properties. Langmuir.

[38] Inamdar DY, Vaidya SR, Mahamuni S (2012). On the Photoluminescence Emission of ZnO
nanocrystals. J Exp Nanosci.

[39] Singh AK, Viswanath V, Janu VC (2009). Synthesis, Effect of Capping Agents, Structural, Optical and
Photoluminescence Properties of ZnO Nanoparticles. J Lumin.

[40] Lin W, Schmidt J, Mahler M, Schindler T, Unruh T, Meyer B, Peukert W, Segets D (2017). Influence of Tail Groups during Functionalization of ZnO
Nanoparticles on Binding Enthalpies and Photoluminescence. Langmuir.

[41] Simmons JG, Reish ME, Foreman JV, Liu J, Everitt HO (2017). How Sulfidation of ZnO Powders Enhances Visible
Fluorescence. J Mater Chem C.

[42] Gheisi AR, Neygandhi C, Sternig AK, Carrasco E, Marbach H, Thomele D, Diwald O (2014). O2 Adsorption Dependent Photoluminescence Emission from Metal
Oxide Nanoparticles. Phys Chem Chem Phys.

[43] Kocsis K, Niedermaier M, Bernardi J, Berger T, Diwald O (2016). Changing Interfaces: Photoluminescent ZnO Nanoparticle Powders in
Different Aqueous Environments. Surf Sci.

[44] Balzar D, Snyder RL, Fiala J, Bunge H (1999). Defect and Microstructure Analysis by Diffraction.

[45] Degen A, Kosec M (2000). Effect of pH and impurities on the surface charge of zinc oxide
in aqueous solution. J Eur Ceram Soc.

[46] Gilbert B, Huang F, Zhang H, Waychunas GA, Banfield JF (2004). Nanoparticles: Strained and Stiff. Science.

[47] Waychunas GA, Zhang H (2008). Structure, Chemistry, and Properties of Mineral
Nanoparticles. Elements.

[48] Zhang H, Gilbert B, Huang F, Banfield JF (2003). Water-Driven Structure Transformation in Nanoparticles at Room
Temperature. Nature.

[49] Zhang H, Banfield JF (2014). Structural Characteristics and Mechanical and Thermodynamic
Properties of Nanocrystalline TiO2. Chem Rev.

[50] Schindler T, Schmutzler T, Schmiele M, Lin W, Segets D, Peukert W, Appavou M-S, Kriele A, Gilles R, Unruh T (2017). Changes within the Stabilizing Layer of ZnO Nanoparticles upon
Washing. J Colloid Interface Sci.

[51] Schindler T, Lin W, Schmutzler T, Lindner P, Peukert W, Segets D, Unruh T (2018). Evolution of the Ligand Shell Around Small ZnO Nanoparticles
During the Exchange of Acetate by Catechol: A Small Angle Scattering
Study. ChemNanoMat.

[52] Deinert J-C, Hofmann OT, Meyer M, Rinke P, Stähler J (2015). Local Aspects of Hydrogen-induced Metallization of the ZnO(1010)
Surface. Phys Rev B.

[53] Stähler J, Rinke P (2017). Global and Local Aspects of the Surface Potential Landscape for
Energy Level Alignment at Organic-ZnO Interfaces. Chem. Phys.

[54] Ozawa K, Mase K (2011). Comparison of the Surface Electronic Structures of H-adsorbed ZnO
Surfaces: An Angle-resolved Photoelectron Spectroscopy Study. Phys Rev B.

[55] Noei H, Qiu H, Wang Y, Muhler M, Wöll C (2010). Hydrogen Loading of Oxide Powder Particles: A Transmission IR
Study for the Case of Zinc Oxide. ChemPhysChem.

[56] Meyer B, Marx D, Dulub O, Diebold U, Kunat M, Langenberg D, Wöll C (2004). Partial Dissociation of Water leads to Stable Superstructures on
the Surface of Zinc Oxide. Angew Chem Int Ed.

[57] Traeger F, Kauer M, Wöll C, Rogalla D, Becker H-W (2011). Analysis of Surface, Subsurface, and Bulk Hydrogen in ZnO using
Nuclear Reaction Analysis. Phys Rev B.

