# Supplementary material for: From Anhydrous Zinc Oxide Nanoparticle Powders to Aqueous Colloids: Impact of Water Condensation and Organic Salt Adsorption on Free Exciton Emission
Source: Langmuir. Author manuscript; Available in PMC 2020 Sep 4. (PMC7116045; doi:10.1021/acs.langmuir.9b00656)
Supplement: Supplementary information [file EMS94066-supplement-Supplementary_information.pdf]

Supporting Information

From Anhydrous Zinc Oxide Nanoparticle Powders to Aqueous Colloids: Impact of Water Condensation and Organic Salt Adsorption on Free Exciton Emission

Krisztina Kocsis[a], Matthias Niedermaier[a], Vít Kasparek[b], Johannes Bernardi[c], Günther Redhammer[a], Michel Bockstedte[a], Thomas Berger[a] and Oliver Diwald[a*]

[a] Department of Chemistry and Physics of Materials,

University of Salzburg, Jakob-Haringer-Strasse 2a, 5020 Salzburg, Austria.

[b] Central European Institute of Technology, Brno University of Technology, Purkynova 123, 612 00 Brno, Czech Republic.

[c] University Service Centre for Transmission Electron Microscopy, Technische Universität Wien, 1040 Vienna, Austria

E-mail: oliver.diwald@sbg.ac.at

Keywords: nanoparticle powders, water adsorption, strain release, free exciton emission, interface engineering;

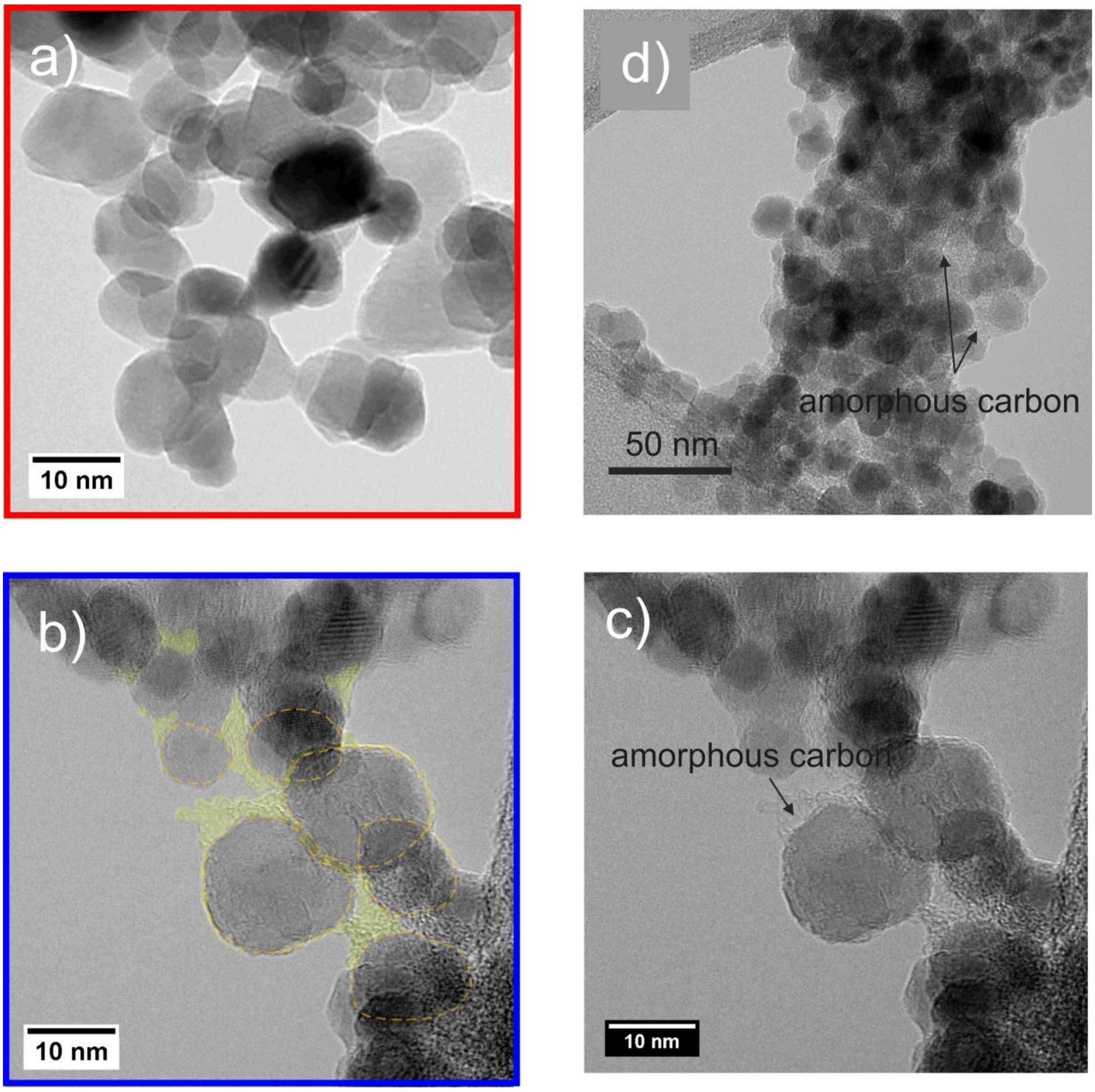

Figure S1: TEM images of vapor phase grown ZnO nanoparticles (a) before and (b) after contact with an aqueous citrate solution. The region in (b) that is highlighted with yellow colour indicates amorphous and carbon-based surface features around the ZnO nanoparticles. These result from adsorbed organics after vacuum drying and electron beam damage. They were observed for all analyzed sample spots of the citrate functionalized nanoparticles, but were not observed in samples that were exclusively processed in vacuum and oxygen. Figures 1c and d show unprocessed images for reference and comparison.

Microstrain analysis

Lattice strain in nanoparticles is a measure of the distribution of lattice parameters arising from their confined volume. It originates from synthesis related defects inside the lattice such as vacancies, interstitials and dislocations. The distribution of strain in nanocrystals is usually assessed with advanced scattering techniques (coherent X-ray diffraction), pair distribution function analysis as well as extended X-ray absorption fine structure (EXAFS) experiments.[1] Here we performed laboratory XRD measurements and subjected the obtained diffraction patterns to refinement using the double-Voigt approach.[2] As crystallite size and microstrain convolutions vary in 2θ as a function of 1/cos(θ) and tan(θ), one can separate these contributions from each other using data that are acquired up to sufficient high 2θ angles.

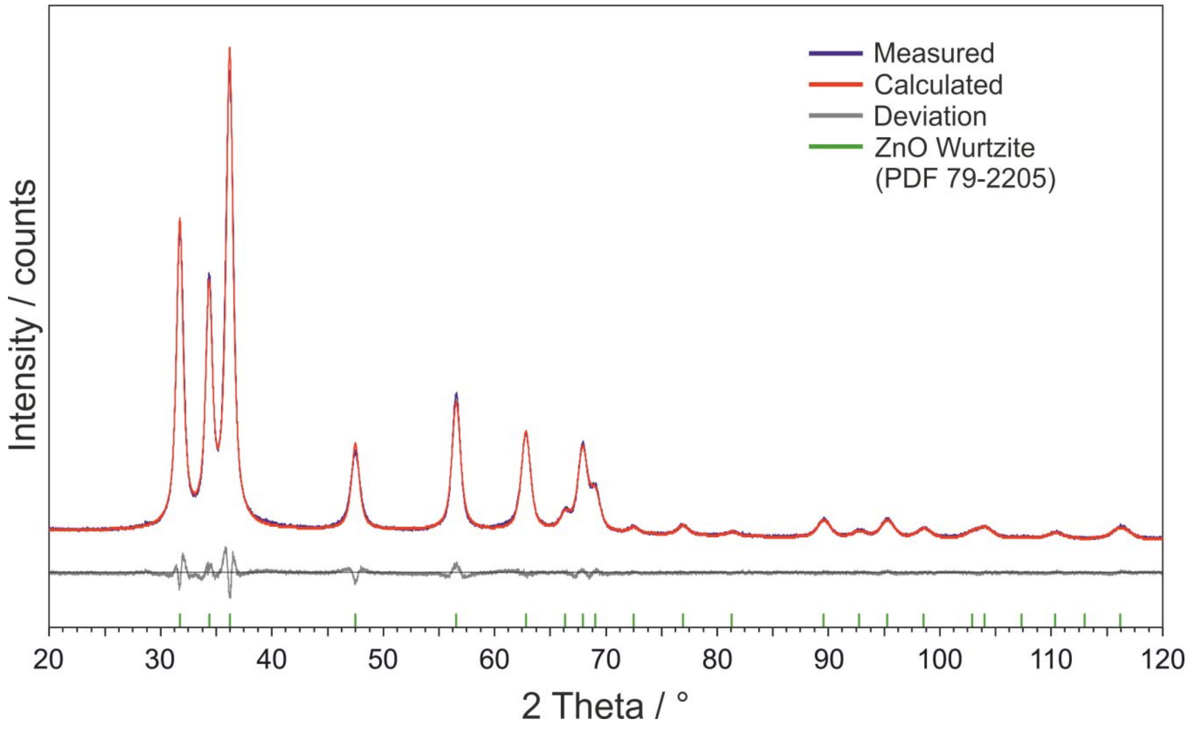

Figure S2: X-ray powder diffraction pattern of an oxidized ZnO nanoparticle powder analysed using the Rietveld method (full pattern refinement).

We found that as-synthesized nanoparticle powders exhibit the largest microstrain, which becomes relaxed in the course of subsequent thermal activation, e.g. from ε = 0.385(10) to ε =

0.225(6). A small internal lattice strain relaxation was determined for oxidized ZnO nanocrystals (measured in Ar atmosphere) and those exposed to air and stored therein, respectively. While extended air exposure (over a period of ~ 7 weeks) does not affect the sample's average crystallite domain size and the microstrain within the margin of the standard deviations estimated, ZnO nanoparticle powders in contact with liquid water (with or without organic acids) exhibit a measurable reduction in microstrain (Table S1).

Whereas the as-synthesized nanoparticles exhibit the largest microstrain, subsequent thermal activation of the powder leads to a substantial relaxation, e.g. from ε = 0.385(10) to ε = 0.225(6). However, a small internal lattice strain relaxation was still determined for oxidized ZnO nanocrystals (measured in Ar atmosphere) and those exposed to air and stored therein, respectively. While extended air exposure (over a period of ~ 7 weeks) does not affect the sample's average crystallite domain size and the microstrain within the margin of the estimated standard deviations, ZnO nanoparticle powders in contact with liquid water (with or without organic acids) exhibit a measurable reduction in microstrain (Table S1). This reduction in strain, e.g. from ε = 0.226(6) to 0.198(4), is observed for thermally activated samples, which serve as a starting material of this study. Even if low, the extent of strain reduction exceeds the estimated standard deviation by a factor of 4 and is observed for both as-synthesized ZnO nanoparticle powders, as well as for those after thermal activation and subsequent contact with liquid water. Even if the absolute values of strain vary from one sample to the other to some extent, the relative trends are entirely the same (Table S1).

Classical and quantum molecular dynamics simulations have indicated that disorder and structural deviations in nanoparticles relative to the bulk material may pervade from the surface throughout nanoparticles.[3] It was shown, that even at ambient temperatures, nanoparticles undergo substantial transformation in structure that is driven by surface

interactions. This indicates that apart from its size dependence internal strain also depends upon the nature of the surroundings.[4,5]

Sample	LVol-IB* (nm)	strain
ZnO nanoparticles oxidized (ID# 143)	9.27(11)	0.251(9)
ZnO nanoparticles in aqueous dispersion	9.63(8)	0.228(6)
ZnO nanoparticles in aqueous and citrate containing dispersion	9.64(9)	0.242(11)
ZnO as-synthesized (ID# 150) nanoparticles	7.169(10)	0.337(1)
ZnO as-synthesized in contact with liquid water	8.361(8)	0.279(9)
ZnO nanoparticles in aqueous and citrate containing dispersion	7.972(8)	0.292(9)
ZnO as-synthesized	6.6(7)	0.385(10)
ZnO nanoparticles oxidized	9.36(6)	0.225(6)
ZnO nanoparticles in aqueous dispersion (ID#153)	10.48(6)	0.198(4)
ZnO nanoparticles in aqueous and citrate containing dispersion	10.12(6)	0.212(4)

Table S1: Particle size, strain and strain release effects observed for ZnO nanoparticles in as-synthesized form, after oxidative treatment at T = 673K and subsequent contact with liquid water. *LVol-IB: volume averaged column height calculated from the integral breadth

Strain and Photoluminescence

It is well established, that strain can affect the electronic and optical properties of ZnO nanostructures. As demonstrated for thin ZnO films supported on different substrates, the lattice mismatch between ZnO film and substrate induces strain and alters the band structure of the semiconductor.[6] On hydrothermally grown ZnO nanorods grown along the c-axis and perpendicular to the substrate, as another example, the near band edge emission significantly increases from as-grown structures to those obtained after subsequent annealing in oxygen atmosphere showing enhanced crystallinity.[7] Moreover, polycrystalline ZnO films grown on sapphire substrates[8] show an increase of the UV PL emission feature after heat treatment, which was linked to the partial elimination of strain in the films using independent diffraction experiments.

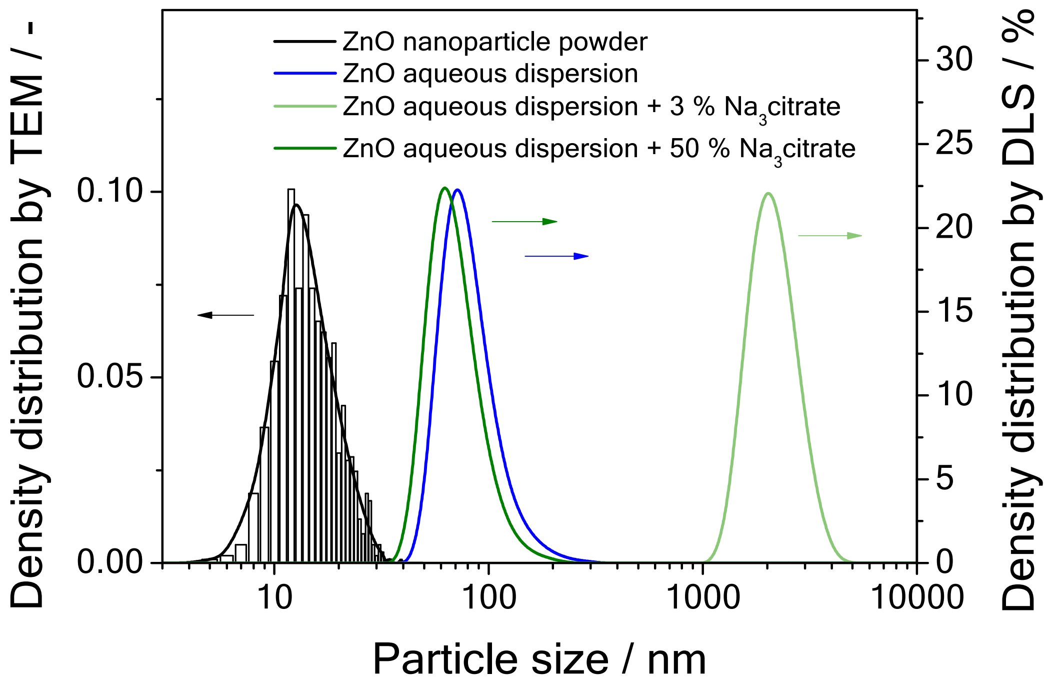

Figure S3: Size distribution plots of primary and secondary ZnO particles. The particle size significantly increases at a relative citrate concentration of 3 m %. Here, the net surface charge approaches a value of zero.

Na_3citr. conc.	d_H/ nm	Zeta potential/ mV
0 %	~70	23.9 ± 0.6
0.5 %	~80	17.0 ± 0.9
1%	~120	10.6 ± 3.4
2 %	~1800	4.6 ± 1.2
3 %	~2000	-1.6 ± 2.0
5 %	~240	-7.3 ± 1.3
8 %	~80	-17.0 ± 3.0
10 %	~70	-19.0 ± 1.7
20 %	~60	-22.4 ± 0.6
30 %	~70	-24.8 ± 0.1
50 %	~60	-25.8 ± 1.7

Table S2: Sodium citrate concentrations, hydrodynamic diameters of the secondary particles (agglomerates) and zeta-potential values.

$ZnAc_2$ conc.	d_H/ nm	Zeta potential/ mV
0 %	~70	23.9 ± 0.6
5 %	~75	27.9 ± 0.2
10%	~80	28.9 ± 0.9
15 %	~65	29.3 ± 0.9
20 %	~55	31.3 ± 0.9
30 %	~70	30.3 ± 0.3

Table S3: Zinc acetate concentrations, hydrodynamic diameters of the secondary particles (agglomerates) and zeta-potential values.

References

(1) Gilbert, B.; Huang, F.; Zhang, H.; Waychunas, G. A.; Banfield, J. F. Nanoparticles: Strained and Stiff. *Science* **2004**, *305*, 651–654.

(2) Snyder R. L.; Fiala J.; Bunge H.; Eds. *Defect and Microstructure Analysis by Diffraction: Voigt Function Model in Diffraction-Line Broadening Analysis*; Oxford University Press: Oxford, 1999.

(3) Zhang, H.; Banfield, J. F. Structural Characteristics and Mechanical and Thermodynamic Properties of Nanocrystalline TiO_2. *Chem. Rev.* **2014**, *114*, 9613–9644.

(4) Zhang, H.; Gilbert, B.; Huang, F.; Banfield, J. F. Water-Driven Structure Transformation in Nanoparticles at Room Temperature. *Nature* **2003**, *424*, 1025–1029.

(5) Waychunas, G. A.; Zhang, H. Structure, Chemistry, and Properties of Mineral Nanoparticles. *Elements* **2008**, *4*, 381–387.

(6) Piyadasa, A.; Wang, S.; Gao, P.-X. Band Structure Engineering Strategies of Metal Oxide Semiconductor Nanowires and Related Nanostructures: A Review. *Semicond. Sci. Technol.* **2017**, *32*, 73001.

(7) Babikier, M.; Wang, J.; Wang, D.; Li, Q.; Sun, J.; Yan, Y.; Wang, W.; Yu, Q.; Jiao, S.; Gao, S.; Li, H. Effect of Annealing on Lattice Strain and Near-Band-Edge Emission of ZnO Nanorods. *Electron. Mater. Lett.* **2014**, *10*, 749–752.

(8) Wang, G.; Zhang, G.; Ketterson, J. B.; Gatt, R. Enhanced Photoluminescence from Polycrystalline ZnO Films Resulting from Oxygen Processing. *Thin Solid Films* **2004**, *460*, 232–236.
